# Trismus during tracheal extubation as a complication of general anaesthesia – A case report

**DOI:** 10.1515/med-2022-0573

**Published:** 2022-10-28

**Authors:** Paweł Radkowski, Bartosz Kędziora, Justyna Dawidowska-Fidrych

**Affiliations:** Department of Anaesthesiology and Intensive Care, Regional Specialist Hospital in Olsztyn, Olsztyn, Poland; Department of Anaesthesiology and Intensive Care, Faculty of Medicine, Collegium Medicum University of Warmia and Mazury in Olsztyn, Olsztyn, Poland; Department of Anaesthesiology, Intensive Care and Emergency Medicine, Hospital Zum Heiligen Geist in Fritzlar, Fritzlar, Germany; Department of Paediatrics, Pro-Medica Hospital in Ełk, Ełk, Poland

**Keywords:** succinylcholine, cannot ventilate, general anaesthesia, bradycardia, laryngeal mask

## Abstract

Tracheal extubation is a risky phase of anaesthesia. Most complications that occur when an endotracheal tube is removed are of minor nature, but those that require critical action can end in serious complications or even death. Patient – A 55-year-old woman was admitted for elective transabdominal hysterectomy and adnexal procedures. Anaesthesia – Standard monitoring. For induction, we used fentanyl, propofol, and rocuronium. The maintenance phase of anaesthesia was without complications. After extubation, the patient presented with severe trismus and mask ventilation was unsuccessful (cannot ventilate) – It was not until 200 mg of succinylcholine was administered that the masseter muscle spasm subsided.

Extubation is a process that must always be planned. A routine approach and lack of a contingency plan is responsible for a number of complications related to the period of patient awakening and associated with the removal of the endotracheal tube. Trismus, in response to extubation, is a phenomenon not described in the literature in non-high risk patients.

## 1 Introduction

Extubation, or the removal of the endotracheal tube in anaesthesia, is usually an uncomplicated procedure and, consequently, its complications are underestimated. Extubation failure requires as much attention as unexpected difficult intubation, especially since as many as 1/3 of serious incidents that involve securing the airway with an endotracheal tube occur at the end of mechanical ventilation [1]. Most complications after extubation involve the respiratory system and irritation of the glottis or bronchial tree. Coughing and respiratory distress, laryngospasm, decreased saturation, bronchospasm, or post-obstructive pulmonary oedema may be observed [2]. Trismus occurs due to a motor disturbance of the trigeminal nerve, especially spasm of the masticatory muscles (the temporalis, masseter, medial pterygoid, and lateral pterygoid muscles), with difficulty or complete inability to open the mouth (lockjaw). This incident is practically absent in the literature as an unexpected complication of extubation. The following clinical case report provides information on what to look for in a critical situation in anaesthesiology such as sudden, unexpected trismus leading to a situation of inability to open the mouth completely.

## 2 Case report

March 2, 2022. A 55-year-old woman was admitted to the Gynaecology Department for an elective transabdominal hysterosalpingo-oophorectomy [excision of the uterus, uterine tubes, and ovaries for hysteromyomas and atypical squamous cells]. The patient had been treated for depression, bipolar affective disorder, and hypertension with paroxetine (30 mg), alprazolam (2.5 mg), and hypertension polytherapy of amlodipine (5 mg), indapamide (1.25 mg), and perindopril (4 mg). She reported an allergy to co-trimoxazole (trimethoprim/sulfamethoxazole). Prior to surgery, intravenous cefazolin (2.0 g) was administered. During the patient’s hospitalisation, additional single doses of the following drugs were administered: subcutaneous enoxaparin sodium (40 mg), intravenous paracetamol (1 g), and oral metamizole (3 × 1.0 g).

The following day, the patient was brought to the operating room. After initiating perioperative monitoring with the following, electrocardiography, non-invasive blood pressure, and haemoglobin saturation, we performed an epidural block at L2/L3 placing a catheter 5 cm deep. Then, we passively oxygenated the patient with 100% oxygen through a face mask for 3 min. For induction of anaesthesia, we used 0.1 mg fentanyl, 150 mg propofol, and 50 mg rocuronium. We succeeded to intubate the patient on the first try using a 7.5 mm tube. Anaesthesia was maintained with a mixture of desflurane and nitric oxide(i), minimum alveolar concentration = 0.9–1.0 in air with fraction of inspired oxygen = 0.5, fresh gas flow = 1 L min^−1^. Lung ventilation was performed in volume mode with positive end expiratory pressure (PEEP) = 5. We administered a drip infusion of 1,000 mL multi-electrolyte fluid. At 50 min after intubation, to optimise analgesic treatment, we administered an epidural bolus of 10 mL of 0.125% bupivacaine and fentanyl, and set the above mixture at a flow rate of 6 mL h^−1^.

Approximately 60 min after intubation, the gynaecologist noticed the first signs of resolving muscle relaxation in the form of single diaphragmatic movements. Accordingly, we administered a bolus of 20 mg rocuronium. The operation ended 85 min after intubation. Before turning off the gas supply (desflurane and nitrous oxide), we injected 200 mg of sugammadex to reverse the neuromuscular blocking drugs. After observing the patient’s own efficient breathing and a positive cough test, we removed the tracheal tube, achieving 100% saturation in the first minute. After this time, the patient suddenly developed a very strong trismus preventing active ventilation on the mask and a critical situation in anaesthesiology emerged – “cannot ventilate.” We immediately administered 150 mg of propofol without a relaxation effect on the masticatory muscles. Consequently, the saturation dropped rapidly to 20% (which means that the accuracy of the measurement was indeterminate) and the heart rate (HR) to 40 beats  min^−1^ with no response to 0.5 mg of atropine. When the HR slowed to 28 beats min^−1^, this prompted us to administer 1 mg epinephrine (systolic pressure = 80 mmHg). Only a bolus of maximum dose of succinylcholine 200 mg broke the contraction of the muscles responsible for the trismus. We promptly placed a No. 4 laryngeal mask, which allowed ventilation and, consequently, an increase in saturation. The patient was protected from other airway complications with 200 mg of theophylline in a continuous drip infusion. Re-extubation with a laryngeal mask was uncomplicated. The patient scored 10 on the Aldrete scale upon awakening. The postoperative period was without complications, and the patient was discharged home 3 days after the surgery.

Using the Naranjo adverse drug reaction probability scale, we calculated a score of (−2) (a score of <0 indicates the reaction was likely related to factors other than a drug) [3]. We also found no reports of interactions between drugs taken chronically by the patient and drugs used throughout the anaesthetic process.


**Informed consent:** Published with the written consent of the patient.

## 3 Discussion

Patients at risk of difficult extubation include those with difficult airway conditions, e.g., injuries to the glottis, larynx, trachea, or with tumour masses in the mediastinum. Special attention should also be paid to obesity, respiratory, and haemodynamically unstable patients as well as patients whose surgery requires the Trendelenburg or “prone position” (when the patient lays face downward) [4]. In the consensus on the use of neuromuscular monitoring in the perioperative period, an international panel of experts pointed out the need for quantitative monitoring of neuromuscular conduction whenever non-depolarizing relaxants are administered. Many national anaesthesia societies support this recommendation in their guidelines [5]. In the described case, the patient was not provided with neuromuscular monitoring due to the lack of availability of a suitable device in the operating theatre. The probability that the neuromuscular blockade was effectively reversed was determined on the basis of: clinical manifestations (spontaneous breathing of the patient, reflex reactions of the larynx to the endotracheal tube, opening the eyes); the fact that 25 min had passed since the repeated dose (20 mg) of rocuronium; and sugammadex doses of 200 mg.

Possible complications of extubation include sore throat, laryngeal oedema, or even vocal cord paralysis. Persistent opiates or neuromuscular blocking drugs should always be excluded. Upper airway obstruction is most commonly caused by collapse of the tongue, and the presence of mucus or blood in the mouth. Searching the literature, which was carried out using the most popular search engines of medical records (PubMed and Google Scholar), provided information that a postextubation complication in the form of severe trismus had ever been described only in patients with craniofacial pathologies, tetanus symptoms, or intoxication.

An increase in muscle tone is an important symptom in anaesthesiology because it directly heralds the onset of malignant hyperthermia after succinylcholine administration (this pathognomonic symptom occurs in approximately 65% of patients). A repeated injection of succinylcholine does not generally remove muscle tension. However, in our case, trismus did not occur in response to a depolarizing muscle relaxant and there were no other symptoms characteristic of malignant hyperthermia such as, for example, increased skin heat, tachycardia, ventricular arrhythmias, or macular skin lesions.

The validity of the administration of succinylcholine when trismus occurred may be controversial, because this drug has many side effects and is currently used very rarely. However, succinylcholine is the fastest and most potent muscle relaxant, the administration of which allows for ventilation in a critical situation in which we observe very low saturation values. It should also be mentioned that the administration of succinylcholine immediately after trismus occurred could also be associated with contraction of the facial muscles through the mechanism of short-term stimulation of the acetylcholine receptors of the motor plate, leading to its depolarization. Another solution would be to administer rocuronium only in a large dose (min 0.9 mg per kg/body weight) to achieve the fastest possible muscle relaxation. However, in this case, one should remember about possible drug interactions with the previously administered sugammadex, which has an elimination half-life (*t*_1/2_) of about 2 h, as well as the consequence of a much longer duration of action of rocuronium compared to succinylcholine [6]. Administration of a high dose of rocuronium would result in a longer time to wake the patient or require a high dose of sugammadex, which is still a very expensive drug.

## 4 Conclusion

- Extubation is a standard procedure in an anaesthesiologist’s practice that is mostly uncomplicated.
- In the case of trismus, the only effective treatment is the administration of muscle relaxants.
- It is necessary to always think about using a laryngeal mask when problems with extubation arise.
